# Cell Death by Metals: Diversity of Phenomena vs. Distinctive Categories

**DOI:** 10.3390/cancers16223767

**Published:** 2024-11-08

**Authors:** Alexander A. Shtil, Kirill V. Chernov, Sergey A. Tsymbal

**Affiliations:** 1 Blokhin National Medical Research Center of Oncology, 24 Kashirskoye Shosse, 115522 Moscow, Russia; 2 International Institute of Solution Chemistry and Advanced Materials Technologies, ITMO University, 9 Lomonosov Street, 191109 Saint-Petersburg, Russia; chernov@scamt-itmo.ru (K.V.C.); zimbal@scamt-itmo.ru (S.A.T.)

Dear Editor:

We were thrilled to read the study by A. Mohapatra et al. entitled ‘Inorganic nanomedicine–mediated ferroptosis: A synergistic approach to combined cancer therapies and immunotherapy’ (*Cancers* 2024, 16, 3210. doi:10.3390/cancers16183210) [1]. This comprehensive and timely review raised questions that deserve a special analysis. We believe that the discussion of the current knowledge of cell death mechanisms would strengthen the basics of eukaryotic cell biology, in particular, fundamental carcinogenesis, and advance the search for rational therapy.

The authors dissected ferroptosis, a complex response of cells to the exogenous iron-containing nanoformulations [1]. It is stated that ferroptosis is a unique mode of cell death due to a number of specific molecular events different from the patterns of apoptosis and other mechanisms. This biological particularity of ferroptosis is regarded as an important feature for combining ferroptosis-inducing agents with conventional chemotherapeutics and immunotherapy. The key mechanistic role in ferroptotic cell death is attributed to iron-dependent generation of reactive oxygen species (ROS) that lead to lipid peroxidation (Figure 1, [1]).

ROS are the cytocidal moieties whose detrimental effects on the structure of cellular macromolecules have been undoubtedly documented [2]. The lipid bilayers in the plasma membrane and cytoplasmic organelles are particularly sensitive to ROS-induced oxidation, a modification that specifies a non-repairable damage of these barriers followed by cell death. The authors integrated the necrotic component of iron-induced death into the entire ferroptotic pathway. In our opinion, this holistic viewpoint is critical for understanding the complex and genuinely intermingled origin of cell death phenomena. The following considerations illustrate our interest and concerns.

First, is ferroptosis a particular cell death mechanism or a peculiar combination of events that generally occur in dying cells? The positive answer presumes the uniqueness of molecular determinants detectable in ferroptosis but not in, say, apoptosis or necrosis. However, ROS generation and subsequent lipid peroxidation represent a part of the cytotoxic stress response, not at all limited to ferroptosis. These species are formed in cells treated with a variety of agents (doxorubicin as an example of a ROS-generating chemotherapeutic drug). In the case of metals, ROS are not restricted to iron-containing agents. Production of oxygen radicals in the Fenton reaction is mechanistically associated with copper (II) reduction to monovalent Cu^+^ cations [3,4]. Recently, cuproptosis has been shown to be a mode of cell death in response to copper-containing compounds [5]. In our experiments ROS-mediated cell death upon electrochemical reduction of Cu^2+^ to Cu^+^ was manifested as an early (within the initial hours) loss of the plasma membrane integrity detectable by cell permeability for propidium iodide, a marker of necrosis [4]. Furthermore, mitochondria were damaged. Concomitantly, their transmembrane electric potential decreased, further substantiating the role of membrane-directed ROS effects. It remains to be elucidated whether mitochondrial dysfunction by exogenous ROS led to secondary (endogenous) oxidation and formation of a self-sustainable ‘ROS circuit’ to ensure cell death. Apparently, the events initiated by a plethora of chemically unrelated compounds, including metals, converge to ROS generation as a common (that is, not stimulus-specific) death effector shared by many treatments. Admittedly, the biochemical alterations in cells treated with metal-containing compounds have individual features such as glutathione depletion and lipid peroxidation for iron nanoparticles [1] or lipoylation of the key tricarboxylic acid cycle enzymes and disturbance of Fe-S cluster proteins for inducers of cuproptosis [5]. Still, in specific cell types the variations dictated by the origin of the metal (and, therefore, associated with differential primary targeting in the cell) may evoke non-identical death mechanisms [6]. Furthermore, the types of death depend on metabolic conditions. Indeed, copper-containing compounds induce cuproptosis given that oxygen respiration is sufficient, so the lowered rate of oxidative phosphorylation can switch cuproptosis to apoptosis [5]. However, this holds true for perhaps any stimulus! What makes ferroptosis special is the manifold events triggered by (or associated with) the excess iron content, namely, iron-induced ROS generation/lipid peroxidation. Convergence of this diversity to relatively narrow (although not necessarily identical in different cell types) number of death execution pathways or their combinations is an inevitable stage shared by other pathways.

Second, the necrotic component (i.e., loss of the integrity of membranes), either it precedes or follows other death mechanisms such as caspase activation, poly(ADPribose) polymerase cleavage or DNA fragmentation, marks the lethal cell damage. In this sense, the principal dichotomy ‘apoptosis vs. necrosis’, in which the plasma membrane integrity is the key discriminant, is critically important. From the practical position, the compounds that induce ferroptosis as well as other ‘metalloptotic’ cell death emerge as treatments of choice for circumventing multifactorial drug resistance in tumor cells survived after repeated courses of chemotherapy. In these cells many apoptotic pathways are no longer functional. Nevertheless, cells remain sensitive to intracellular ROS burst or to the exogenous oxidants, indicating that, even in pleiotropically resistant cells, the necrotic mechanism is still operational. Thus, metal-containing drugs offer valuable opportunities for treatment of advanced tumor disease. In particular, given that multidrug transporters are frequently overexpressed in drug resistant cells, the intracellular concentrations of conventional chemotherapeutics can be insufficient for ROS formation inside the cells. However, metal-containing compounds may act in the extracellular milieu to generate ROS outside the cells upon Cu^2+^ reduction [4]. In this scenario the loss of the plasma membrane integrity is decisive for the cell’s fate regardless of whether other death cascades are or aren’t activated.

These considerations bring us to the general problem of the diversity of cell death mechanisms. The ‘apoptosis vs. necrosis’ dichotomy discriminates the two major mechanisms claiming the plasma membrane integrity as a key criterion. This division underlies the clear practical need for the selection of adequate therapeutic approach. For instance, it is worth exploiting an inducer of a necrotic-like death in situations when apoptotic pathways are non-functional, and their engagement for treatment is problematic [7]. Therefore, the above dichotomy seems fully justified. But is the variety of events that precede the plasma membrane damage meaningful enough to designate a distinctive death mechanism? Does the list of types of non-necrotic cell death deserve expansion along with the growing roster of inducers, keeping in mind the manifold origin of exogenous cues and the variability of primary biological responses? In other words, one and the same iron-containing compound can trigger early events characteristic for ferroptosis but the involvement of individual mechanisms of death execution, i.e., caspase activation or mitochondrial damage or DNA fragmentation or altered plasma membrane permeability, as well as their ordering, would depend, at least in part, on the cell type. We tend to state that, for a death mechanism to be proved as distinctive, the molecular phenomena in treated cells must be unique rather than comprise a network of events common for many stimuli. Definitely, the number of death mechanisms should not prevail over the imaginable amount of cytotoxic cues!

We are grateful to the authors of the publication [1] for an opportunity to formulate our argumentation. Hopefully, the presented thoughts will raise valuable criticism from the readership of *Cancers.*
